# An urban medical system's exploratory study of medication errors

**DOI:** 10.1002/nop2.319

**Published:** 2019-06-17

**Authors:** Skip G. Morelock, Jeffrey D. Kirk

**Affiliations:** ^1^ Collin College School of Nursing McKinney Texas; ^2^ Independent statistician

**Keywords:** medication errors, nurses, nursing

## Abstract

**Aims:**

This study sought to identify patterns of medication errors with respect to shifts, day of week, unit involved, severity, medication class and cause of errors and to propose possible solutions.

**Design:**

This was a retrospective explorative study using a database containing 605 medication events from two medical centres. Variables assessed include medication type, the error severity, and time the medication was ordered, the unit that the error occurred on and the day of the week of the errors.

**Methods:**

Simple percentages were used to report the results, and point‐biserial correlation was employed to test for significant differences between the day and night shifts.

**Results:**

There were no statistically significant findings when comparing event severity against the a.m. or p.m. shifts. The medication classes with the most errors were antibiotics, and the most common reason cited for errors was dose omission. The most commonly reported severity level was a 2 which requires increased patient monitoring.

## 1 INTRODUCTION

The definition of a medication error is “any preventable event that may cause or lead to inappropriate medication use or patient harm while the medication is under control of the healthcare professional…” (National Coordinating Council for Medication Error Reporting & Prevention, 2016).

Despite recent advances in patient medication administration including the introduction of computerized order entry, electronic patient identification and medication barcode scanning, medication errors continue to occur. These systems were designed to drastically reduce and even eliminate medication errors at the nurse–patient interface and if explicitly followed, should have resulted in zero errors. As often occurs with innovation, the problem of unintended consequences arises. Solutions which seem to make perfect sense in the developing stage often do not translate seamlessly to the imperfect and unpredictable clinical world. Since the problem of medication administration errors continues, it makes sense to explore why and more importantly, isolate and formulate practical and operational solutions. The identification of particularly problematic classes of drugs is key and may catalyse the development of more focused solutions. Nurses will remain the central figure as they are usually the final stop between the medication and the patient. They are the final barrier. The goal of this research was to examine medication errors and to gain insight into the where, when and why they are continuing to occur despite continued emphasis on safe patient passage.

## 2 BACKGROUND AND LITERATURE REVIEW

With the increasing focus on nurse sensitive metrics and their concomitant impact on outcomes and Centers for Medicare and Medicaid Services (CMS) reimbursements, it follows that all aspects of nursing practice are subject to intense scrutiny. Carlton and Blegen (2006) examined medication errors through the lens of quality management and categorized medication errors as either active or latent. Both categories represented errors which reached the patient. An active error was caused by dose miscalculation and dose omission while latent errors were promoted, at least in part, by extraneous environmental factors such as fatigue, interruptions during the medication procurement and administration process and staffing issues. Other causes of latent errors seem to be influenced by poor nurse educational preparation or lack of basic pharmacological knowledge (Bower, Jackson, & Manning, 2015; Frith, Anderson, Tseng, & Fong, 2012). Emphasizing and managing conditions which may lead to errors can be highly complex mainly because one cannot control for or eliminate all intrinsic or extrinsic factors which have an impact on medication administration. In 2012, Frith et al. conducted a retrospective correlational study to determine the effect that staffing variables and skill mix have on medication errors. It was found that the more complex the medical diagnosis, the more likely it was that the patient would experience a medication error during their hospitalization. Tzeng, Yin, and Schneider (2013) reported that 9.2% of all admitted people will experience an adverse event related to medication administration. While that number seems very high, it is likely significantly below the actual number. For reasons ranging from inattentional blindness to the desire to avoid disciplinary action, many nurses do not report or even recognize medication errors. These same researchers also identified that medication administration incidents represented 53% of all reported clinical errors. Of these, 58% were medication doses that were missed and 21% because the wrong dose was administered (Tzeng et al., 2013). Other notable takeaways from this study include the finding that decreasing the frequency of licensed practical nurse (LPN) use resulted in an overall decrease in errors. This finding held for all inpatient nursing units where LPNs were used. The effect of complexity of diagnosis and increased errors has been found in other research (Breckenridge‐Sproat, Johantgen, & Patrician, 2012; Hall, Doran, & Pink, 2004).

The issue of nurse interruptions while administering medications continues to surface in contemporary nursing research. Studies have linked interruptions during the medication administration process with increased errors (Bower et al., 2015; Harkanan, Turunen, Saano, & Julkenen, 2013; Malone, 2016). More ominously, Raban and Westbrook (2014) found that errors committed because of interruptions were more likely to result in serious patient harm and death. These researchers identified three types of interruptions which impinge on the nurse's time and ultimately affect the process of medication delivery. The first are sudden and unpredictable changes in the patient's condition which merit rapid intervention and may interrupt the medication administration process mid‐stream. This type of interruption makes it difficult to maintain the ordered timeliness of medication delivery since the nurse will have to manage the existing emergency and then go back to the patient and either initiate the medication administration process all over again or return in the middle of the process, thus increasing the chance that a critical step will be omitted. In one study, nurses were interrupted a average of 43 times in a ten‐hour period during medication rounding (Tucker & Spear, 2010). Other studies showed that 17% of all medication administrations were interrupted in some way (Young et al., 2015). It is unreasonable to think that this type of interruption can be completely eliminated, but it can be reduced.

Another type of interruption occurs when well‐meaning families insist on persistently asking the nurse about the patient when the nurse is trying to calculate or titrate the dose of a drug or intravenous medication. This problem may be especially prevalent in critical care units with an open visitation policy and where powerful vasoactive medications are being titrated. Family members may not even be aware that this type of interruption can cause a nurse to commit a potentially dangerous medication error. Some hospitals have implemented a system where if a nurse is at the medication dispensing machine, calculating a medication, titrating or adjusting an intravenous drip, or is in the patient/medication scanning phase of the medication administration process, they are not to be interrupted (Bower et al., 2015; Bravo, Cochran, & Barrett, 2016; Cloete, 2015). While this sounds workable in theory, operationally it might be difficult to implement effectively and consistently. Interventions which purport to reduce interruptions during the medication administration process have not been shown to be very effective (Raban & Westbrook, 2014). Additionally, hospitals might be slow to implement any actions which seem to discourage people or families from asking questions.

The third type of interruption occurs when a physician, nurse, other provider or staff member interrupts the nurse to relay insignificant or inconsequential information that may even be unrelated to the patient that is being care for. Tucker and Spear (2010) found that when physician's interrupted nurses during medication administration to relay redundant messages such as “I have written new orders” often had the effect of making the nurse feel frustrated and demeaned as they are well‐aware of the need to check for new orders and the procedure for checking new orders is hardwired into their daily practice. This interruption will also have the same effect as other interruptions since the nurse will have to re‐attend to the original task and creating the possibility of an error.

Since medication errors can happen at any time, it is prudent to examine any potential differences between hospital work shifts. The nurses in this health system continue to work 12‐hr non‐rotating shifts. The perception of this organization was that nursing care and nursing quality on the night shift and possibly on weekends might be compromised because of the increased use of per diem and external agency staff.

## 3 ETHICAL CONSIDERATIONS

The health system's Institutional Review Board (IRB) approved the research prior to data collection. This body also determined that the study met all ethical requirements of the National Institutes of Health. Since the examination of medication errors could have potentially uncovered sensitive information with a possible impact of career about the nurses involved in these misadventures, there was no information collected which could reasonably be used to identify the nurses involved.

## 4 METHODS

This research focused primarily on the nurse–patient interface and the errors that occur at this point in the process, the actual administration of the medication. While the author fully recognizes that medication errors can (and do) occur at every part of the medication administration cycle, the nurse is the final barrier between the medication and the patient and arguably represents the most critical element of the process.

This study is best described as a retrospective exploratory study involving two urban medical centres in north Texas. One of the hospitals is a Magnet© designated facility while the other facility has Pathways to Excellence© recognition. The combined bed capacity is approximately 650. The larger facility offers solid organ transplant services and the other facility, though smaller, functions as a Level III trauma centre. Medication error reports were collected for the time period of 1 January 2016–31 July 2017 across all of the site hospitals inpatient units and perioperative areas. This research seeks to discover either latent or obvious error commonalities and to probe the data for levels of harm incurred from the misadventures and also if any work shifts were particularly problematic with respect to either errors or enhanced severity.

Variables assessed were departments where errors occurred, classification of medications implicated in the errors, administration issues, administration routes of ordered medication, time and shift that the errors occurred, day of the week of the event and the assessed severity of the errors. Nominal level variables include the medication class, department, shift, mode of administration and day of the week that the errors were committed. The one continuous level variable is the event severity. Severity Level 1 means that there was no harm to the patient and no further intervention was required. Severity Level 2 requires increased monitoring of the patient. An error that is coded as Severity Level 3 means that the patient not only required an increase in monitoring, but also required additional time in the hospital. This level also includes an upgrade in the level of care. Severity Level 4 means that there was temporary harm to the patient while Level 5 event, while relatively rare, means that the patient will have some degree of permanent harm as a result of the error. Severity Level 6 is reserved for deceased people who, after extensive chart review, have died with the event in question as the primary or major contributing factor which leads to the death.

Quality department abstractors at each facility, via each medical centre's event reporting system, identified all pertinent medication events for the research time frame. The data were then scrubbed of any patient identifying information as well as the names of individual nurses involved which helped ensure little to no patient, staff risk or that individual harm would result from the study. To preserve data integrity and ensure uniformity, only one abstractor at each facility was tasked with identifying and pulling medication error events from the event reporting system. The scrubbed data, redacted of identifiable protected health information, were delivered electronically to the author for database creation and subsequent analysis.

The final data set comprises 605 medication events that reached the patient. Near misses, although important, were not used in this analysis since they did not represent an actual drug administration error. Results were reported by category using simple percentages. Point‐Biserial correlation was used to test for significant differences between event severity and the a.m. and p.m. shifts.

## 5 RESULTS

See Table 1 for the tabular representation of the results. The category of Severity Level 1 was seen in 26.8% of the data set. Severity Level 2 accounted for 46.1% of the events and represented the most common occurrence outcome. The remainder of the events were classified as Severity Level 3 at 24% and Severity Level 4 at 3.0%. Severity Level 5 accounted for 0.3% of the sample. There were no deaths (Severity Level 6) associated with any of the medication events.

**Table 1:** Charts and graphs (*N* = 605)

Variables								
Severity magnitude	Severity Level 1 (162) 26.8%	Severity Level 2 (279) 46.1%	Severity Level 3 (145) 24.0%	Severity Level 4 (18) 3.0%	Severity Level 5 (2) 0.3%	Severity Level 6 (0) 0.0%		
Errors by drug class	Antibiotics (121) 20.0%	Intravenous Cardiac (102) 16.9%	Analgesic (80) 13.2%	Anti‐Neo (66) 10.9%	Total parental nutrition/electrolytes/IVs (66) 10.9%	Anti‐rejection (36) 6.0%	Anti‐coagulants (33) 5.5%	Gastrointestinal medication (32) 5.3%
	Insulins (22) 3.6%	Labour‐regulating (15) 2.5%	Anxiolytics/ Hypnotics (11) 1.8%	Misc. (11) 1.8%	Anti‐hypertensives (4) 0.7%	Contrast media (4) 0.7%	Steroids (2) 0.3%	
Reason for error	Omitted (138) 22.8%	Late (132) 21.8%	Overdosed (126) 20.8%	Early (106) 17.5%	Wrong route (67) 11.1%	Wrong drug (20) 3.3%	Monitoring error (10) 1.7%	Wrong patient (6) 1.0%
Route of administration	Intravenous (462) 76.4%	Oral (104) 17.1%	Subcutaneous (30) 5.0%	Topical (5) 0.8%	Intramuscular (2) 0.3%	Intrathecal (1) 0.2%	Inhaled (1) 0.2%	
Day of the week	Monday (91) 15.1%	Tuesday (87) 14.3%	Wednesday (93) 15.4%	Thursday (72) 12.0%	Friday (108) 17.8%	Saturday (69) 11.4%	Sunday (85) 14.0%	
Unit that error occurred	Acute care (299) 49.4%	Critical care (186) 30.7%	Oncology (82) 13.6%	Labour and delivery (15) 2.5%	Neonatal intensive care (11) 1.8%	Emergency department (8) 1.3%	Perioperative areas (4) 0.7%	
Errors by shift	a.m. (359) 59.3%	p.m. (246) 40.7%						

With an extensive pharmacopeia available to people at the site facilities, medications were allocated into 14 distinct categories and a 15th category for miscellaneous medications that did not conveniently fit into the other categories. This grouping allowed for better examination and analysis of the data. Three medication groups, Intravenous Cardiac Medication (16.9%), analgesics (13.2%) and antibiotics (20%), accounted for 50.1% of all reported medication errors.

Regarding the specific issues involved in medication errors, the most commonly reported error was an omitted dose of an ordered medication (22.8%), while late administration (21.8%) and overdoses (20.8%) accounted for much of the remainder of events. When errors were examined based on day of the week, there was a fairly equal distribution of events across the days. Fridays logged the most errors at 17.8% while Saturdays accounted for the least percentage of errors at 11.4%. Results also show that intravenous (76.4%), oral (17.1%) and subcutaneously administered medications (5.0%) were the most commonly cited routes in these errors. Intramuscular, intrathecal, inhaled and topical routes of administration only accounted for a combined 1.5% of the total.

Errors were also categorized by the type of unit where they occurred. Critical care and acute care units were responsible for 80.1% of the total number of errors. Oncology units accounted for 13.6% of the total with significantly lesser percentages logged in the L&D, perioperative, emergency and neonatal intensive care areas.

**Graph 1:**
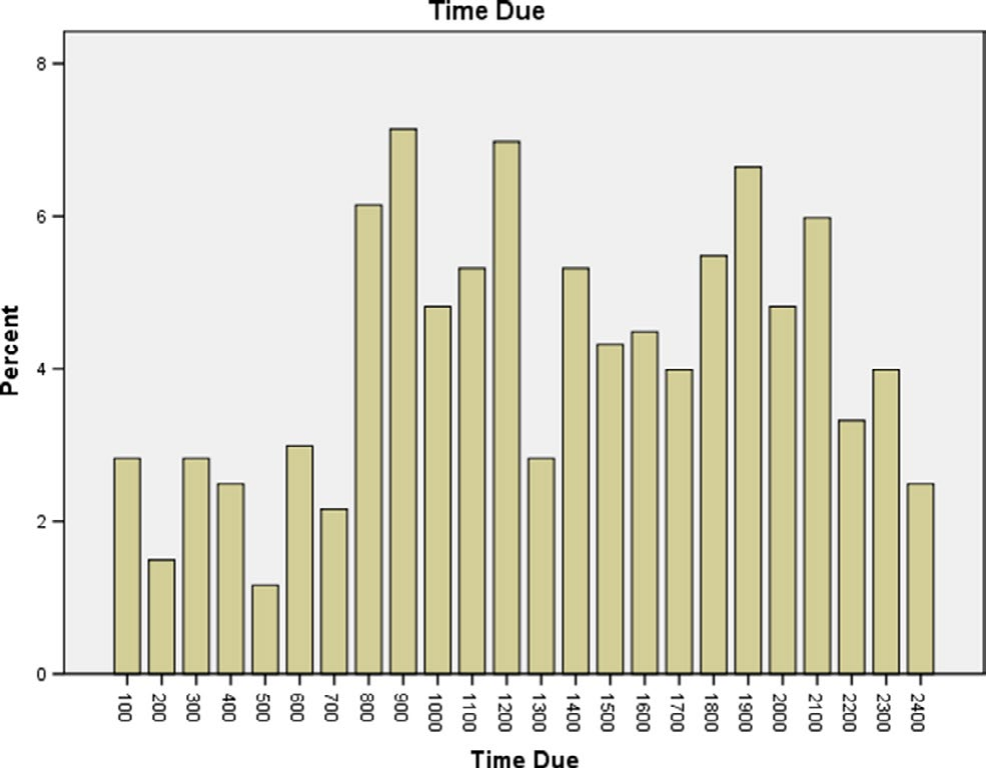
Ordered medication times

The researcher also captured the time that the ordered medication was due to be administered (Graph 1). The histogram displays a diffuse bimodal distribution of the data points with peaks roughly corresponding to 09:00 and 20:00.

Finally, a Point‐Biserial correlation was conducted using shift as the dichotomous variable (Table 2). There were no statistically significant findings in Event Severity between the two shifts. The mean magnitude was 2.6 for the a.m. shift versus 2.5 for the p.m. shift.

**Table 2:** Point‐Biserial correlational analysis

	Shift occurred	Event severity	
Shift occurred	Pearson correlation	1	−0.012
	Sig. (2‐tailed)		0.761
	*N*	605	605
Event severity	Pearson correlation	−0.012	1
	Sig. (2‐tailed)	0.761	
	*N*	605	605

There were notable differences between the a.m. and p.m. shifts that were revealed during this research (Table 3). IV Cardiac medication (a.m. shift 15.0%/p.m. shift 19.5%), GI medication (a.m. shift 3.9%/p.m. shift 7.3%) and insulin (a.m. shift 2.8%/p.m. shift 4.9%) logged more errors on the p.m. shift than the a.m. shift despite more opportunities for administration during the a.m. shift. Regarding units where the errors occurred, the critical care units showed a significant difference in errors between the a.m. and p.m. shifts (a.m. shift 27.0%/p.m. shift 36.2) The p.m. shift, in this current study, also had more incidents of omitted medications (a.m. shift 20.9%/p.m. shift 25.6%) and late administration of medications (a.m. shift 19.2%/p.m. shift 25.6%).

**Table 3:** a.m. and p.m. Shift comparison

Variables	a.m./p.m.							
Severity magnitude	Severity 1 25.3%/28.9%	Severity 2 47.1%/44.7%	Severity 3 25.3%/22.0%	Severity 4 2.2%/4.1%	Severity 5 0.0%/0.4%	Severity 6 0.0%/0.0%		
Errors by drug class	Antibiotics 20.9%/13.4%	Intravenous cardiac 15.0%/19.5%	Analgesic 13.1%/13.4%	Anti‐neoplastics 12.3%/8.9%	Total parenteral nutrition/electrolytes/IVs 10.6%/11.4%	Anti‐rejection 7.0%/4.5%	Anti‐coagulants 5.8%/4.9%	Anti‐hypertensives 0.3%/1.2%
	Gastrointestinal medication 3.9%/7.3%	Insulin 2.8%/4.9%	Labour‐regulating 2.8%/2.0%	Anxiolytics/Hypnotics 1.7%/2.0%	Misc. 2.5%/0.8%	Contrast 1.1%/0.0%	Steroid 0.3%/0.4%	
Reason for error	Omitted 20.9%/25.6%	Late 19.2%/25.6%	Overdosed 21.7%/19.5%	Early 17.5%/17.5%	Wrong route 13.4%/7.7%	Wrong drug 3.9%/2.4%	Monitoring 2.2%/0.8%	Wrong patient 1.1%/0.8%
Route of administration	IV 75.2%/77.6%	Oral 17.8%/15.9%	SQ 5.3%/4.5%	Topical 0.6%/1.2%	IM 0.6%/0.0%	Intrathecal 0.3%/0.8%	Inhaled 0.3%/0.0%	
Day of the week	Monday 15.6%/14.2%	Tuesday 15.9%/11.8%	Wednesday 14.8%/16.3%	Thursday 12.0%/11.8%	Friday 18.4%/16.7%	Saturday 10.6%/13.4%	Sunday 12.8%/15.9%	
Unit that error occurred	Acute care 50.4%/48.0%	Critical care 27.0%/36.2%	Oncology 15.9%/10.2%	Labour and delivery 3.1%/1.6%	Neonatal intensive care 1.9%/1.6%	Emergency department 0.8%/2.0%	Perioperative areas 0.8%/0.4%	

## 6 DISCUSSION

Error magnitude is of particular importance since any error has the potential to result in permanent sequelae or death. There are also financial implications with regard to medication errors. Extended lengths of stay (LOS), changes in the level of patient care (acute care to intensive care) and the payment of compensatory damages can adversely affect a hospital's operational budget and public reputation.

Somewhat surprising are that errors involving analgesics (which includes Schedule II opioids) accounted for only 13.2% of the total errors. The researchers surmised prior to beginning the research that this would be considerably higher. This less than expected finding may be a result of the increased scrutiny being placed on the availability and suitability of this class of drugs for analgesia during and following routine procedures and for short‐term pain control as well as increased institutional and regulatory oversight.

Interestingly, drug administration to the wrong patient accounted for only 1.0% of the total. This is an encouraging finding as earlier studies exploring medication errors put this figure at closer to 6% (Hughes & Blegen, 2008; Mohammed, Human, Esmaeil, & Syyedeh, 2013). It may be that the Joint Commission's (TJC) continued emphasis on accurate patient identification is finally becoming hardwired into a routine culture of safety in the medication administration process instead of being regarded by the bedside nurse as another cumbersome step which had to be completed and which was frequently seen as expendable.

An examination of the errors when compared with the day on which they occurred shows no meaningful difference on the weekends. This was a somewhat unexpected finding since the perception among the administrative staff and the hospital's coordinating councils was that more errors occurred on weekends as there is less administrative oversight. In this study, that perception was not supported.

The relatively low percentage of errors reported in the emergency department (1.3%) in this study was unexpected. Research has shown that emergency departments, because of rapidly changing volumes, unit churn and a mixture of high and low patient acuity, should have a high rate of medication misadventures when compared with other hospital units (Ehsani et al., 2013; Vazin, Zamani, & Hatam, 2014). The authors suspect that medication misadventures in this clinical area may be underrepresented in this particular study.

While examining the individual events, it became apparent that all of the reports involving contrast administration were because of severe adverse reactions to the media and not from an overdosing. The reactions ranged from wheezing and difficulty breathing to anaphylaxis requiring the administration of subcutaneous and racemic epinephrine to control. Also of heightened interest were the events involving intrathecal administration of medicaments. Though rare, both documented incidences were scored as either Level 4 or Level 5 in severity. Based on this study, the protocols were modified so any ordered intrathecal medications are hand delivered by a licensed pharmacist to the nurse of record and the dose validated and signed off by another Registered Nurse prior to administration.

The graph that represents when the medications involved in the errors were due to be give provides some clarity about when the errors are occurring. These times also happen to be when many routine medications are timed in the hospital setting. Of additional interest is that on the p.m. shift, the 19:00 time also shows a peak in administration time errors. This is also the time that most hospitals undergo a change of shift. This may represent an artefact of the data, or it may be reflective of the evidence that errors seem to occur more frequently during the changeover period from day shift to night shift (Mardis et al., 2016). A similar spike is not seen at the time of the 0700 shift change.

The lack of statistically important differences with regard to severity when comparing shifts was not anticipated. There were, however, important differences between the shifts when examining certain categories of medications. Of particular interest are the apparent higher incidents of omitted medications on the p.m. shift and the higher percentage of occurrences of events involving intravenous cardiac medications. These differences may be promulgated, at least in part, by a nurse's natural reticence to not awaken sleeping people and practicing in a darkened environment. Fatigue may also be playing a role in these observed differences. It has been known for some time that night shift nurses scored lower on psychomotor tests than their day shift counterparts (Johnson, Brown, & Weaver, 2010). Higher errors and severity of errors have also been linked to new or novice nurses who may not have the experience or clinical knowledge required to manage highly complex people. It is still the practice of these facilities to place all novice nurses on the night shift for a period of time. In a recent study, Morelock (2016) found that critical care nurses who had less than two years of clinical experience and worked the p.m. shift had a significantly higher incidence of error commission (*r* = −0.31; *p *= 0.037).

## 7 RECOMMENDATIONS

Three proposed recommendations for improvement will be discussed in this section. The first is that unnecessary interruptions during medication administration rounds must be significantly curtailed. A variety of measures have been attempted to ensure that the nurse preparing and administering medications can do so with as few interruptions as possible. Some include lighted lanyards which alert staff, visitors and people that the nurses are on medication rounds and should not be unnecessarily interrupted, the “sterile cockpit” approach has been tried by some organizations with limited success (Kapur, Parand, Soukup, Reader, & Sevdalis, 2015), medication preparation areas can be marked or cordoned off and only permit one nurse at a time to enter and procure medications (Hayes, Jackson, Davidson, Daly, & Power, 2017), and some have adopted a strategy where the nurse who is giving medication dons a brightly coloured vest to denote that medication rounding is occurring (Johnson et al., 2017). Unfortunately, all of these methods may promote a sense, at least from people and families that the nurses should never be interrupted. Impairing communication with the healthcare team or people may be an unintended consequence of these implementations and impeding communication with any member of the healthcare team would not promote a desirable outcome. Other approaches include the silencing all intrahospital communication devices during the medication preparation and administration processes and routing incoming personal calls through the unit clerk for vetting prior to transferring any call to the nurse. In many organizations, overhead paging has been curtailed or even eliminated altogether in an effort to create a quieter, more restful and less frenetic clinical environment.

The second recommendation is that barcoding of people and medications be enacted in every patient care area where medications are procured and administered. While by no means a panacea, implementation of barcoding is becoming standard in most hospitals (Mekonnen, Abebe, McLachlan, & Brien, 2016). Initially, there had been considerable concern that the process of barcoding would take up an inordinate amount of time and could possibly harm people if the nurse or physician was not able to access emergency drugs in a rapid and expeditious manner. In fact, these problems did occur with the 1st generation of barcoding technology in that it would sometimes take several minutes to reconcile and “load” into the automatic medication dispensing machine or convey the medication to the nurse (Poon et al., 2008). In response, nurses would develop workarounds to ensure that emergency medications were always available. There were also concerns that people might view barcoding as unnecessary or too personally invasive or that the nurse, already strapped for time, would now spend even more time away from their people to scan and reconcile medications. Yet, when barcode scanning is consistently practiced and the supporting technology platform is robust and accurate, the result is that fewer medication errors occur (Seibert, Maddox, Flynn, & Williams, 2014; Truitt, Thompson, Blazey‐Martin, NiSai, & Salem, 2016).

The third recommendation is somewhat controversial, but is important to broach and discuss. The notion of a completely non‐punitive culture of safety, while of the best intent, may be indirectly contributing to medication errors. When the non‐punitive approach to reporting medication errors became more widespread during the early 21st century, it was of great importance since it allowed real‐time data to be obtained and made it possible to root out process obstacles which had been contributing to medication errors. It was important that nurses felt comfortable in reporting all medication errors including the near misses. Unfortunately, this seems to have evolved from a “duty to report with the understanding that there might be consequences, that the patient is better served by honest disclosure” to “it is not really the nurses fault if errors are made if there are process issues that are broken, inefficient or need modification.” The idea that nurses can absolve themselves of all responsibility for medication administration errors may be in direct conflict with many Boards of Nursing position statements and practice acts. In Texas, nurse managers or those that manage nurses and nursing practice must track all clinical errors committed by nurses (including medication errors) and report either to the Board or to the entities peer‐review committee, a commission of greater than five errors in any 12‐month period, if a troubling pattern of errors occurs, or if there is permanent harm, injury or death because of an error (TX BON‐ rule 217.16). Hospitals are not doing bedside nurses any favours when they claim that there will be no consequences for nurses by self‐reporting their errors. This may be instilling an attitude of complacence and a lessening of the sense of duty and responsibility that nurses must imbue and embrace as they care for people. That said, nurses must continue to report errors that they commit or observe since they are ethically and legally required to do so. Nursing schools should make it abundantly clear to their graduating students that that they are ultimately responsible and accountable for medications that they administer to a patient. Nursing leaders and nursing administrative staff should instruct the nurses that report to them about the requirements of an individual state's board of nursing mandatory error reporting, if any and to ensure compliance, explain that errors may be tracked and trended. The recommendation therefore is to inform and emphasize to all nurses that they are ultimately responsible and accountable for their own practice and that errors committed by them should be viewed pragmatically and that nursing leadership should seek to remove barriers to safe nursing care while still ensuring that the nurse remains accountable for their practice and to their people.

## 8 STRENGTHS AND WEAKNESSES

Studies of this nature are difficult to analyse using statistically robust techniques such as ANOVA and logistic regression. This limits the ability to infer the results to other organizations. The information presented, however, is important in that it shows how errors are distributed in this hospital system and also provides future direction for research. It also catalysed the nursing leaders in this organization with impetus to continue to proactively root out causes for medication errors. Additionally, it garnered important information on how errors trended as the work week progressed and quelled suspicions that the level of medication errors was markedly different on weekends and on the p.m. shift. Future research will help refine and focus on the continuing problem of medication administration errors.

## 9 CONCLUSION

As administrators and leaders, it is incumbent on us to act in a way that ensures an increased level of patient safety. The discussion of drug administration errors has assumed a very important place in contemporary healthcare dialogue. Systems that are designed to reduce errors have not proven to be completely successful and errors are still occurring. This “first look” at a hospital systems effort to more carefully study drug administration errors of omission and commission has revealed interesting trends as well as actionable data which have been brought to the bedside. There is no existing universal solution, and there is no reason to think that any system can be developed that is able to exclude the intrinsic human factors in medication administration. The mere act of seeking solutions to these problems will certainly unearth more focused opportunities for improvement. Nurses on the front lines of medication administration must be open to learning new strategies and adapting new ways to practice more safely. The overarching goal is for there to be no reason for any patient or people’ loved one to be fearful about a medication mistake being made during a routine hospital admission.

## CONFLICT OF INTEREST

There was no conflict of interest in any phase of this research.

## AUTHOR CONTRIBUTION

Dr. Morelock was responsible for designing the study and the preparation of the manuscript. Mr. Kirk assisted in the statistical analysis and in the interpretation of the results.
